# Using height-for-age differences (HAD) instead of height-for-age z-scores (HAZ) for the meaningful measurement of population-level catch-up in linear growth in children less than 5 years of age

**DOI:** 10.1186/s12887-015-0458-9

**Published:** 2015-10-06

**Authors:** Jef L. Leroy, Marie Ruel, Jean-Pierre Habicht, Edward A. Frongillo

**Affiliations:** Poverty, Health, and Nutrition Division, International Food Policy Research Institute, 2033 K Street NW, Washington, DC 20006 USA; Division of Nutritional Sciences, Cornell University, Savage Hall, Ithaca, NY USA; Arnold School of Public Health, University of South Carolina, Columbia, SC USA

**Keywords:** Catch-up growth, Linear growth retardation, 1000 days, Children

## Abstract

**Background:**

Evidence from studies conducted in nutritionally deprived children in low- and middle-income countries (LIMC) in past decades showed little or no population-level catch-up in linear growth (mostly defined as reductions in the absolute height deficit) after 2 years of age. Recent studies, however, have reported population-level catch-up growth in children, defined as positive changes in mean height-for-age z-scores (HAZ). The aim of this paper was to assess whether population-level catch-up in linear growth is found when height-for-age difference (HAD: child’s height compared to standard, expressed in centimeters) is used instead of HAZ. Our premise is that HAZ is inappropriate to measure changes in linear growth over time because they are constructed using standard deviations from cross-sectional data.

**Methods:**

We compare changes in growth in populations of children between 2 and 5 years using HAD vs. HAZ using cross-sectional data from 6 Demographic and Health Surveys (DHS) and longitudinal data from the Young Lives and the Consortium on Health-Orientated Research in Transitional Societies (COHORTS) studies.

**Results:**

Using HAD, we find not only an absence of population-level catch-up in linear growth, but a continued deterioration reflected in a decrease in mean HAD between 2 and 5 years; by contrast, HAZ shows either no change (DHS surveys) or an improvement in mean HAZ (some of the longitudinal data). Population-level growth velocity was also lower than expected (based on standards) in all four Young Lives data sets, confirming the absence of catch-up growth in height.

**Discussion:**

We show no evidence of population-level catch-up in linear growth in children between 2 to 5 years of age when using HAD (a measure more appropriate than HAZ to document changes as populations of children age), but a continued deterioration reflected in a decrease in mean HAD.

**Conclusions:**

The continued widening of the absolute height deficit after 2 years of age does not challenge the critical importance of investing in improving nutrition during the first 1000 days (i.e., from conception to 2 years of age), but raises a number of research questions including how to prevent continued deterioration and what is the potential of children to benefit from nutrition interventions after 2 years of age. Preventing, rather than reversing linear growth retardation remains the priority for reducing the global burden of malnutritionworldwide.

## Background

Chronic malnutrition in children remains an important global problem, with an estimated 165 million children under five being stunted [1]. Evidence suggests that the most effective way to reduce stunting globally is to scale-up interventions to *prevent* (rather than *treat* or *reverse*) stunting, and that children should be exposed to these interventions during the full first 1000 days of life (from conception to the child’s second birthday) [1–3]. This period is now universally recognized as the “window of opportunity for preventing undernutrition” and nutrition programs increasingly target women and children during this critical period. This programmatic shift from the earlier focus on children under 5 years of age has been implemented not only because of the recognition that this is the period of most rapid growth failure [4], but also because there is some evidence, albeit mostly from one country (Guatemala), that interventions beyond this age have little or no impact on linear growth [5]. Thus, a common view in the nutrition community is that linear growth retardation is largely irreversible after two years of age, when the window of opportunity for improving nutrition has closed.

Despite the consensus achieved on the importance of the first 1000 days, the verdict on the potential for catch-up in linear growth during mid- or later-childhood and at adolescence remains open. The term *catch-up growth* was first used to describe the reversal of linear growth retardation in individual children treated for secondary growth disorders such as renal disease, Cushing’s syndrome, celiac disease and hypothyroidism [6, 7]. Catch-up growth was defined as “rapid linear growth that allowed the child to accelerate toward and, in favorable circumstances, resume his/her pre-illness growth curve” (in Boersma and Wit [7], p. 646). Adoption studies have also shown that malnourished children adopted into wealthier households during their first few years of life experience substantial catch-up in linear growth. Little or no population-level catch-up growth in height has been found, however, in groups of children who remained in the same deprived settings in which linear growth retardation had occurred in the first place [8].

Notwithstanding these earlier findings, the possibility that linear growth retardation can be (even if only partially) reversed has continued to intrigue researchers. A number of recent studies document population-level catch-up in linear growth after 2 years of age in children exposed to standard of care practices typical of developing country contexts, but in the absence of interventions specifically aimed at improving linear growth [9–11]. By contrast with earlier studies which mostly used reductions in the absolute height deficit [8] at the individual level to define catch-up growth in height, this new body of research uses changes in mean height-for-age z-scores (HAZ) (or in percentage of children who transitioned from being stunted (HAZ < −2) to not stunted (HAZ ≥ −2) over time) to define catch-up growth.

The main objective of this paper was to assess whether there is evidence of population-level catch-up growth in height in children between 2 and 5 years of age when catch-up growth is defined as it was originally, as *a reduction in the absolute deficit in height* (compared to standards) between two points in time. To derive population-level estimates, we use mean height-for-age difference (HAD: child’s height compared to standards, expressed in centimeters) and compare with findings using mean HAZ. The rationale for this comparison is that HAZ, which is constructed using standard deviations from cross-sectional data, is useful to assess children’s attained height at a given age, but inappropriate to evaluate changes in height as children age [12]. We first show mathematically that using HAD to assess catch-up in linear growth is fundamentally different from defining catch-up growth using HAZ. We then use data from several developing countries and compare changes in linear growth and evidence of population-level catch-up growth in height in children between 2 and 5 years of age when estimated using HAD versus HAZ.

## Methods

### Study scope and definition

Catch-up in linear growth can be defined at the individual and population level. For individual children, it is defined as a reduction in the absolute deficit in height (compared to the standards) between two points in time. Catch-up growth in height is only possible when children grow faster than the expected velocity (for their age and sex) so they can make up for the lost growth in height. This paper focuses on population-level catch-up in linear growth, which is defined as a reduction in the mean absolute height deficit as groups of children age.

Most of the recent studies that reported population-level catch-up growth in height looked at changes in mean HAZ between childhood and either adolescence or adulthood [11, 13–15]. Others looked at changes in mean HAZ between early infancy (first 2 years of age) and mid-childhood (e.g. 5–6 years) [9–11]. Our analysis focuses on the latter period, and therefore our research addresses the question of whether or not population-level catch-up in linear growth is achieved between two and five years of age. Given our focus on population-level catch-up growth in height, regression to the mean does not affect our analyses [16]. Regression to the mean is the tendency of individual children selected based on their shorter or taller heights than that of the population to have heights closer to the mean when they are measured a second time.

For simplicity, we use the terms height, height-for-age difference (HAD), and height-for-age z-score (HAZ) irrespective of the child’s age throughout this paper despite that supine length, rather than standing height, is usually measured in children less than 2 years of age and that the terms “length” and “length-for-age” are typically used for these children.

### Theoretical background

Since infants and young children from diverse ethnic groups grow similarly for the first 5 years of life when their nutrition, health, and care needs are met [17, 18], a single international growth standard can be used to quantify the population-level height deficit for the first 5 years of life. The mean growth trajectory of a population of healthy children is expected to be at the median of the growth standards. A population-level height deficit (i.e., mean height being below the median of the standard) reflects growth impairment caused by a deficient environment (i.e., poor diet, inadequate care and poor health) to which the population of children has been exposed. Population-level height deficits are expressed as the mean of the individual deficits. These are calculated as the difference between the measured height and the median sex- and age-specific height obtained from the growth standards. This height-for-age difference (HAD) can be used in absolute terms (as proposed here) or it can be divided by the sex- and age-specific standard deviation to calculate HAZ as is done in the later studies of catch-up growth in height.

### Mathematical background

HAZ is calculated as show in Eq. 1.

1 $$ HAZ=\frac{observed\kern0.36em  height- median\kern0.36em  height\kern0.36em  growth\kern0.36em  standards}{SD}=\frac{height-for- age\kern0.36em  difference}{SD}=\frac{HAD}{SD} $$

**Fig. 1:**
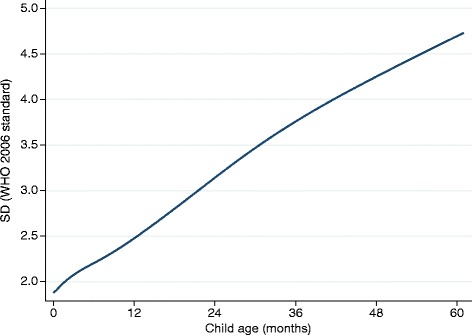
Standard deviation (SD) for height by age (WHO 2006 growth standard)

The SDs for height are not constant over time; they increase substantially from birth to 5 years of age (Fig. 1). Therefore, if HAD is negative but remains constant with age, the Z-score will increase with age (suggesting catch-up growth in height) for the simple, mathematical reason that the denominator (SD) increases, and not because the numerator (the absolute height deficit) has decreased over time.

As noted earlier, most of the recent studies that found evidence of catch-up growth in height based their conclusions on the observation that population mean height-for-age z-score (HAZ) increased after 2 years of age. These studies define population-level catch-up in linear growth as an increase in mean HAZ over time Eq. (2).

2 $$ \begin{array}{l}HA{Z}_{t=2}>HA{Z}_{t=1}\\ {}\kern0.36em \iff \varDelta HAZ>0\end{array} $$

The interpretation of Eq. 2 is that, if HAZ is higher at time 2 than at time 1, there is catch-up growth in height in this population during the time period studied. The validity of this definition of catch-up growth in height is questionable. HAZ are constructed using standard deviations from cross-sectional data and thus provide a useful tool for the assessment of attained growth at one point in time. HAZ is inappropriate, however, to assess changes in height over time and thus to assess catch-up growth in height. Furthermore, changes in HAZ with age can be a consequence of changes in the numerator (the magnitude of the difference, HAD) or of changes in the denominator (the SD increasing with age, see Fig. 1). This makes the change in HAZ across ages difficult to interpret [12].

A more meaningful definition of population-level catch-up in linear growth is a reduction in the mean HAD as a group of children ages:

3 $$ \begin{array}{l}HA{D}_{t=2}>HA{D}_{t=1}\\ {}\kern0.48em \iff \varDelta HAD>0\end{array} $$

Equation 3 requires the actual height velocity to be larger than the expected velocity (i.e., velocity from the growth standard).

The *z-score criterion* Eq. (2) and the *absolute difference criterion* Eq. (3) are fundamentally different as is shown below. Following from Eq. 2, the *z-score criterion* can be written as:

$$ \frac{HA{D}_2}{S{D}_2}>\frac{HA{D}_1}{S{D}_1} $$

(subscripts 1 and 2 refer to t = 1 and t = 2, respectively). We now define *ΔSD* = *SD*_2_ − *SD*_1_. We then get:

4 $$ \begin{array}{l}\kern2.16em \iff \frac{HA{D}_1+\varDelta HAD}{S{D}_1+\varDelta SD}>\frac{HA{D}_1}{S{D}_1}\\ {}\iff S{D}_1\left(HA{D}_1+\varDelta HAD\right)>HA{D}_1\left(S{D}_1+\varDelta SD\right)\\ {}\kern2.16em \iff S{D}_1\varDelta HAD>HA{D}_1\varDelta SD\\ {}\kern2.52em \iff \varDelta HAD>HA{D}_1\frac{{}_{\varDelta SD}}{{}^{S{D}_1}}\end{array} $$

The *z-score criterion* is thus different Eq. (4) from the *absolute difference criterion*, *ΔHAD* > 0. The *z-score criterion* will lead to (erroneous) conclusions of population-level catch-up growth in height when the *absolute difference criterion* does not. The reason is that $HA{D}_1\frac{{}_{\varDelta SD}}{{}^{S{D}_1}}<0$, since *HAD*_1_ is always negative (*HAD*_1_ is a deficit relative to the growth standards) and $\frac{{}_{\varDelta SD}}{{}^{S{D}_1}}$ is always positive (SD increases with age).

Motivated by these theoretical considerations, we compared population-level patterns of growth obtained for children from several developing countries when using changes in mean HAZ versus mean HAD. Statistical testing of HAD versus HAZ results is meaningless because HAZ is a “one to one” sex- and age-specific transformation of HAD.

### Ethics statement

Ethical approval was not required for this analysis of anonymized secondary data. The Demographic and Health Surveys (DHS) data collection procedures were approved by *ORC Macro*’s institutional review board. Each DHS survey was reviewed by the relevant in-country ethics review board. The Young Lives study protocol was approved by the Ethics Committee of Oxford University and by review boards in Ethiopia, India, Peru, and Vietnam. Written informed consent was obtained from participants in all analyzed surveys.

### Datasets

Our analyses used three different types of data. First, we used data from 6 purposefully selected DHS from Latin America (Guatemala, Peru), Africa (Benin, Ethiopia) and South Asia (India and Bangladesh). The countries were selected based on the availability of data sets with large sample sizes. DHS, funded by the U.S. Agency for International Development, are nationally-representative household surveys that collect data on a wide range of population, health, and nutrition indicators. Permission for use of the DHS data was obtained directly from the DHS website (http://dhsprogram.com/data/Access-Instructions.cfm).

Our second source of data is from the Young Lives study which has collected data since 2002 on cohorts of children in Peru, Ethiopia, India and Vietnam, with the intent to track the children for 15 years [19]. We used data for children at the time of enrollment, when children were between 6 and 18 months of age, and at first follow up, when they were between 4.5 and 6 years of age. Permission for use of the YL data was obtained from the UK Data Archive at the University of Essex.

Finally, we redrew a figure from the COHORTS (Consortium on Health-Orientated Research in Transitional Societies) study presented in Stein and colleagues (Fig. 1 in [20]), using mean HAD instead of mean HAZ.

### Data analyses

For approximately 17 % of all children in the DHS datasets used, the day (but not the month or year) of birth were missing. To maximize the number of observations that could be included, a random day of birth was generated for these children. After creating the age in days for all children, we calculated the height-for-age z-scores using the World Health Organization (WHO) 2006 growth standards [21]. The HAD in cm was calculated by subtracting the sex- and age-specific WHO 2006 growth standards median height from the child’s actual height [22]. Observations with an absolute value of HAZ value larger than 5 were dropped from the analyses.

Two types of analyses were conducted using the DHS data. First, we computed mean HAD to compare how they change with age, compared to mean HAZ. We graphed the means of both variables by completed month and the smoothed values using the kernel-weighted local polynomial smoothing algorithm in Stata (version 13.1). Using the smoothed values, we then calculated the change in mean HAZ and HAD by year, i.e. the change from birth to 11 months, from 12 to 23 months of age, and other yearly intervals up to 60 months of age.

Analyses of the Young Lives data were limited to children who were younger than 60 months of age at follow-up and had valid HAZ values (same criterion as above) in both surveys. Observations with an absolute change in HAZ between rounds larger than 4 SD were dropped. As we did for the DHS data, we graphed the mean HAZ and HAD at baseline and follow-up. We calculated changes in HAD and HAZ over time, and compared the observed growth velocity with the expected velocity (i.e., the velocity derived from the WHO 2006 growth standard).

In our final set of analyses, we estimated mean HAD at different ages using published summary statistics from five cohort studies conducted in low- and middle-income countries (see Stein et al. [20]). Mean HAD could not be calculated at mid-childhood for children in the Philippines (96 months is outside the range of the WHO 2006 international growth standards) and South Africa (implausible reported HAZ/height of children 60 months of age).

## Results

The survey country, year, and type, the age range, the total sample size and the number of children included in the analyses are shown in Table 1 for the DHS and Young Lives data sets. Nearly all surveys were conducted since 2000. The percentage of observations that could be included in the analyses varied from around 75 % in Benin to 96 % in Peru.

**Table 1:** DHS and Young Lives data sets analyzed

Type of survey and country	Survey	Age range	Observations included in the analyses	
	(Year)	(Months)	(N)	*(Proportion of total)*
DHS				
Bangladesh	2011	0–59	7635	*0.87*
Benin	2006	0–59	12126	*0.75*
Ethiopia	2003	0–59	9450	*0.81*
Guatemala	1999	0–59	3860	*0.78*
India	2006	0–59	41327	*0.80*
Peru	2012	0–59	9219	*0.96*
Young Lives				
Ethiopia	2002	6–15	520	*0.90*
	2006	55–59		
India	2002	6–18	240	*0.93*
	2006	55–59		
Peru	2002	5–12	393	*0.95*
	2006	53–59		
Vietnam	2002	4–15	332	*0.94*
	2006	50–59		

**Fig. 2:**
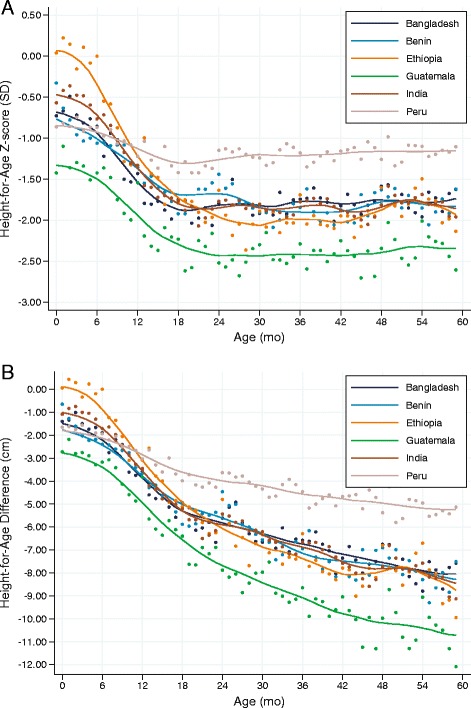
Height-for-age Z-score and height-for-age difference (DHS data). Mean height-for-age z-scores (**a**) and height-for-age difference (**b**) relative to the WHO standard (1 to 59 months) by completed month and kernel-weighted local polynomial smoothed values. Data from *n* = 83,617 children from 6 DHS surveys

Figure 2a shows that substantial growth faltering was present in all 6 DHS countries according to the HAZ. The magnitude of the linear growth retardation, however, differed considerably between countries. Except for Ethiopia, mean HAZ started below the standard with deficits ranging from -0.5 z-scores in India to a very large deficit of around -1.3 z-scores in Guatemala. Mean z-scores then dropped up to 18 to 24 months in all countries, after which they stabilized and slightly increased in some of the countries. The largest drop (around -2 z-scores) was seen in Ethiopia; even though children in Peru started with the second largest deficit at birth (around -0.8 z-scores), the subsequent drop was the smallest of the 6 countries studied (less than 0.5 z-scores), resulting in the highest mean z-score after two years of age. Children in Benin, Bangladesh, and India followed a similar growth pattern: starting with a mean z-score of about −0.50 to −0.75, children stabilized at about −1.8 z-scores after 24 months. Children in Guatemala were by far the worst off with z-scores well below the other countries at all ages, with a mean HAZ after 24 months close to −2.5 z-scores.

Similar to the HAZ curves, the HAD curves (Fig. 2b) showed that the mean absolute height deficit at birth varied considerably across countries: from no deficit in Ethiopia to a massive mean deficit of nearly −3 cm in Guatemala. Also similar to the HAZ curves, the most pronounced faltering (i.e., the steepest slope) was found between 6 and 18 months of age. In sharp contrast with what the HAZ curves suggested, however, substantial growth faltering continued after 24 months of age in all countries, with mean HAD ranging from −5.2 cm in Peru to −10.7 cm in Guatemala. The slopes of the curves provided no indication that the process of growth faltering slowed, which suggests that it might also continue beyond 5 years of age. The “bumps” in Fig. 2a and b just after 24, 36, and 48 months were due to age rounding and heaping, i.e., the tendency to report age in completed years rather than in exact months.

**Fig. 3:**
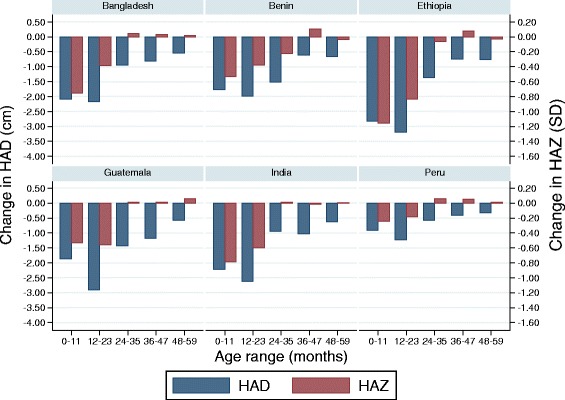
Changes in height-for-age Z-score and height-for-age difference. Mean changes in height-for-age z-scores (red) and height-for-age difference (blue) by year for 6 DHS surveys (1 to 59 months) (n ranges from 3860 to 41,327)

The magnitude of the changes in mean HAZ and HAD during each of the first 5 years is shown in Fig. 3, by yearly age intervals. As would be expected from the previous results, the significant drops in mean HAZ were limited to the first two years of life, and larger in the first compared to the second year for all countries except Guatemala. After two years, there were either no changes, or small increases in mean HAZ. These small increases, however, have led to some of the recent claims of population-level catch-up growth in height after two years of age described in the literature. The changes in mean HAD by year showed a different picture. First, the groups of children lost ground with respect to the standards during every single year of the first 5 years of life, with the largest drops occurring before 24 months of age, and even more importantly during the second year in all 6 countries (drops in mean HAD during the second year of life range from −1.2 cm in Peru to −3.2 cm in Ethiopia).

**Fig. 4:**
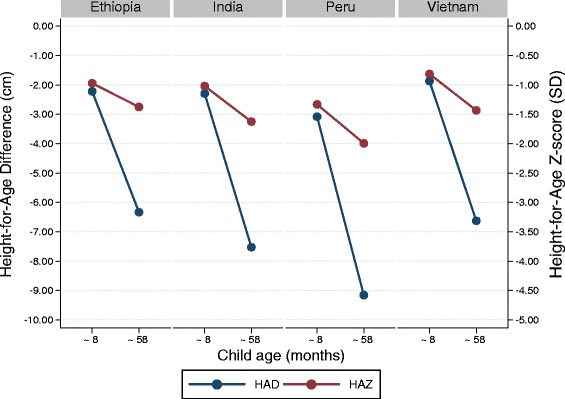
Height-for-age Z-score and height-for-age difference (Young Lives data). Mean height-for-age z-scores (red) and height-for-age difference (blue) at baseline (children around 8 months of age) and follow-up (around 58 months of age) of the Young Lives 4 country cohort study (n ranges from 240 to 520)

Mean HAZ and HAD in the four Young Lives country cohorts are shown in Fig. 4. At baseline, when children were on average 8 months of age, mean HAZ ranged from −0.81 z-scores in Vietnam to -1.33 in Peru. At follow-up (children on average 58 months old), mean HAZ had dropped further in all countries to reach values ranging from −1.38 to −1.99 z-scores in Ethiopia and Peru, respectively. Mean HAD at baseline was around −2 cm for Ethiopia, India, and Vietnam and −3 cm in Peru. At follow-up, the mean absolute height deficit had approximately tripled in all 4 countries. The changes in mean HAZ and HAD between baseline and follow-up are shown in Table 2. Mean HAD dropped in all 4 data sets with the largest drop experienced in Peru (-6.1 cm), followed by India (−5.2 cm), Vietnam (−4.8 cm) and Ethiopia (−4.1 cm). Similarly, observed velocity in height between the two data points was lower than expected velocity in all four data sets, confirming the absence of population-level catch-up in linear growth in these populations. Even among the group of children categorized as having experienced catch-up growth in height according to the *z-score criterion*, linear growth velocity was lower than expected from the standard across all four data sets. Thus even groups of children classified as having experienced catch-up growth in height using the *z-score criterion* grew at a rate slower than the expected rate, and hence accrued additional deficit in absolute height from baseline to follow-up in all countries.

**Table 2:** Change in HAZ, HAD and height from baseline (children around 8 months of age) to follow-up (around 58 months of age) and expected velocity in height in the Young Lives 4 country cohort study by catch-up growth (using the *z-score criterion*)

			Ethiopia	India	Peru	Vietnam
All children		*n*	*520*	*240*	*393*	*332*
		Change from baseline to follow-up				
		HAZ	-0.40	-0.61	-0.66	-0.62
		HAD (cm)	-4.1	-5.2	-6.1	-4.8
		Velocity in height^a^				
		Observed (cm)	34.8	35.1	32.2	34.4
		Expected (cm)	38.9	40.3	38.3	39.1
By catch-up according to *z-score criterion*	No	*n*	*324*	*167*	*308*	*267*
		Change from baseline to follow-up				
		HAZ	-1.27	-1.2	-1.01	-0.92
		HAD (cm)	-6.3	-6.9	-7.3	-5.8
		Velocity in height^a^				
		Observed (cm)	32.6	33.7	31.1	33.7
		Expected (cm)	38.9	40.6	38.3	39.4
	Yes	*n*	*196*	*73*	*85*	*65*
		Change from baseline to follow-up				
		HAZ	1.03	0.75	0.61	0.61
		HAD (cm)	-0.5	-1.5	-1.7	-0.5
		Velocity in height^a^				
		Observed (cm)	38.4	38.3	36.4	37.4
		Expected (cm)	38.9	39.8	38.1	37.9

^a^The observed velocity in height is the mean change in height between baseline and follow-up. The expected velocity is the expected change in height using the WHO 2006 growth standards

**Fig. 5:**
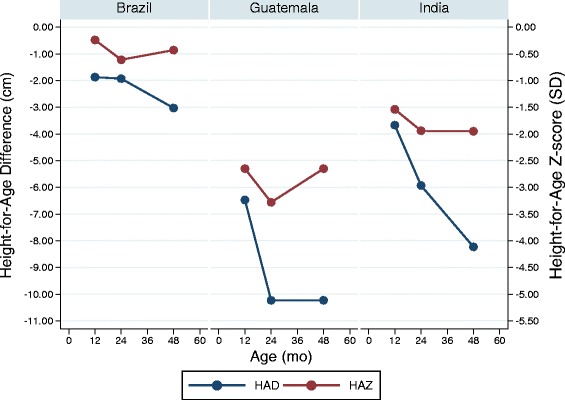
Height-for-age Z-score and height-for-age difference (COHORTS data). Height-for-age z-scores (red) and height-for-age difference (blue) at 12 months, 24 months, and mid-childhood of children in three birth cohort studies (data obtained from Stein et al. [20])

The three COHORTS countries for which mean HAD could be calculated at mid-childhood confirmed that there was no evidence of catch-up growth in height when using HAD (Fig. 5). HAD worsened significantly from 24 to 48 months in Brazil and India and remained stable (and very large > 10 cm) in Guatemala.

## Discussion

Using data from some of the same cohort studies that recently reported population-level catch-up growth in height using the z-score (HAZ) criterion (4 from Young Lives and 3 from COHORTS), we showed not only an absence of population-level catch-up growth in height between 2 and 5 years of age, but a continued deterioration reflected in a decrease in mean HAD. These findings were also supported by population-level linear growth velocity being lower than expected (based on standards) between the two time periods in all four study populations. Catch-up growth in height implies that children grow faster than expected to re-gain lost growth in height, but this was not observed in the data sets analyzed.

Similarly, our analysis of cross-sectional data from DHS surveys showed no sign of catch-up growth in height in the 6 data sets analyzed. Using mean HAZ, we confirmed previous findings of a steep decline in linear growth during the first 18-24 months of age, followed by a leveling off of the curves and an absence of further deterioration up to 60 months of age [4, 12]. By contrast, when using HAD, we showed no sign of improvement or flattening of the curve between 24 and 60 months of age, but rather a continued decline in HAD over time. Based on these findings from analyses of both Young Lives and DHS data sets, we conclude that there is no evidence of population-level linear catch-up growth in these data sets.

Changes in mean HAD, rather than changes in mean HAZ, should be used for the meaningful assessment of population-level catch-up growth in height. HAZ can be used to assess attained growth at a given point in time and allow for comparisons between sex and age groups. HAZ are inappropriate to measure changes in linear growth over time because they are constructed using standard deviations from cross-sectional data. In addition, the definition of HAZ makes it impossible to identify whether changes in HAZ with age are due to changes in the numerator (the magnitude of the deficit) or to changes in the denominator (the increasing SD with age).

Our results do not challenge the assumption that individual- or population-level catch-up growth in height are possible; however, they confirm findings from earlier reviews that population-level catch-up in linear growth does not usually occur among children who remain in the same impoverished environments and are exposed to the same health care, nutrition, and hygiene practices that led to growth faltering in the first place [8]. Given that none of the data sets we used (except for the Guatemala COHORTS data) were from studies that tested the impact of specific nutrition and health interventions among children exposed at different ages, our analysis does not answer the question of whether or not catch-up growth in height beyond 2 years of age in response to successful programs is possible. This question has been answered authoritatively in the Guatemala study, which consistently showed greater benefits from a nutrition and health intervention including a protein-energy supplement on a series of outcomes, including physical, cognitive and economic outcomes in adulthood, among children who were exposed to the intervention before 2–3 years of life, compared to those exposed when they were older [23]. This study, however, has not been reproduced in other countries and it could be that Guatemala is a special case.

As we documented before [12], our finding showing that the accrual of absolute deficits in height continue well into childhood (and possibly beyond) raises an important question related to the timing of the window(s) of opportunity for improving nutrition. Although there is no doubt that the first 1000 days is a critical period for preventing undernutrition, the question of whether or not something can be done to prevent further deterioration beyond 2 years of life remains unanswered. The curves derived from the DHS data are descriptive and do not provide information on the potential to benefit from interventions; the cohort studies (except for the Guatemalan study) were also not designed to answer this question. It is possible that the continued increase in the magnitude of the absolute height deficit between 2 and 5 years is a long-term consequence of inadequate health, nutrition, and care experienced during the first 1000 days, and may or may not be reversible with interventions after 2 years of age. The continued deterioration may also be due to the sustained poor health, nutrition, and care environment to which children between 2 and 5 years of age continue to be exposed.

Many of the recent studies that reported population-level catch-up in linear growth using a HAZ definition focused on changes between early childhood and adolescence or adulthood [11, 13–15]. This requires a different approach than comparing children at different ages within the period of 0-5 years. The reason is that for children < 5 years of age, international growth standards have been developed based on evidence that infants and young children from diverse ethnic groups grow similarly for the first 5 years of life if their nutrition, health, and care needs are met [17, 18]. This evidence, however, does not exist for older children and during adolescence. For the latter, a particular challenge is that malnourished children tend to have a delayed pubertal growth spurt compared to the healthy children included in growth standards (see for instance Kulin et al. [24] and Parent et al. [25]); this makes comparisons with references to quantify height deficits during adolescence difficult to interpret. Other approaches must therefore be developed to measure catch-up growth in height during periods such as adolescence when growth references may not accurately reflect growth potential. We suggested earlier that the possibility of population-level catch-up in linear growth in this age group should be evaluated through experimental studies in which the linear growth of groups of children or adolescents receiving a growth promoting intervention is compared to that of a comparable non-intervention group [26].

Child linear growth is the best available summary measure of chronic malnutrition. Linear growth retardation reflects exposure to a deficient environment (i.e., poor diet, inadequate care and poor health). It also predicts a host of important outcomes throughout the life cycle, including mortality, cognitive development, behavioral outcomes, school achievement, economic productivity and risks of chronic diseases [1]. The importance of these functional correlates, and the potential reversibility of linear growth retardation and related negative functional outcomes, has motivated many of the studies on catch-up growth in height. Whether linear growth retardation is part of the biological causal pathway linking the determinants of malnutrition to these outcomes, however, is not known, nor is the extent to which interventions aimed at improving population-level linear growth –and possibly achieving catch-up in linear growth—can also successfully remedy the functional correlates of linear growth retardation.

## Conclusions

Our analyses using mean HAD found a lack of evidence of population-level catch-up growth in height in cohort studies, and revealed substantial deterioration in absolute height deficit beyond 2 years of age in both cohort and cross-sectional studies. The findings do not challenge the current focus on the first 1000 days as the critical window to improve nutrition. They highlight, however, the need for research to: 1) better understand whether preventing linear growth retardation during the first 1000 days can also help prevent further deterioration in linear growth during mid-childhood and beyond; and 2) identify the types of nutrition inputs that may be needed beyond 2 years of age to at least stabilize, if not reduce the magnitude of the absolute height deficit. Another important question that remains unanswered is whether catch-up growth in height, if possible, results in meaningful reversal of some of the functional consequences of undernutrition in early childhood. New research aimed at elucidating the potential of catch-up growth in height beyond 2 years of age and its consequences on other outcomes, however, should not distract from the current programmatic focus on the first 1000 days and the growing commitment of countries to scale up nutrition interventions (SUN initiative, see http://scalingupnutrition.org/) specifically targeted to mothers and children during the first 1000 days. Research and programming aimed at improving nutrition among adolescent girls and young women before pregnancy and identifying platforms to deliver these interventions at scale remain important too. Preventing undernutrition, rather than reversing it, should continue to be the key goal for tackling the global burden of malnutrition.

## Abbreviations

COHORTS: Consortium on Health-Orientated Research in Transitional Societies
DHS: Demographic and Health Surveys
HAD: Height-for-age difference
HAZ: Height-for-age z-score
LIMC: Low- and middle-income countries
SUN: Scaling up Nutrition
WHO: World Health Organization
